# Geospatial analysis in addressing surgical care inequities: A scoping review and methodological guide

**DOI:** 10.1371/journal.pdig.0001510

**Published:** 2026-07-16

**Authors:** Marc Cohen, Rui Fu, Jonathan Irish, Julie Hallet, Frances Wright, Natalie Coburn, Peter Gozdyra, Abdel-Rahman Lawendy, Antoine Eskander

**Affiliations:** 1 Evaluative Clinical Sciences, Sunnybrook Research Institute, Toronto, Ontario, Canada; 2 Departments of Community Health Sciences, Surgery & Oncology, Cumming School of Medicine, University of Calgary, Alberta, Canada; 3 Institute of Health Policy, Management, and Evaluation, University of Toronto, Toronto, Ontario, Canada; 4 Princess Margaret Cancer Centre, Toronto General Hospital, Department of Otolaryngology-Head and Neck Surgery/Surgical Oncology, University Health Network, Toronto, Ontario, Canada; 5 Department of Otolaryngology – Head and Neck Surgery, University of Toronto, Toronto, Ontario, Canada; 6 ICES, Toronto, Ontario, Canada; 7 Division of Surgical Oncology, Odette Cancer Centre – Sunnybrook Health Sciences Centre, Toronto, Ontario, Canada; 8 Department of Surgery, Sunnybrook Health Sciences Centre, Toronto, Ontario, Canada; 9 Department of Surgery, University of Toronto, Toronto, Ontario, Canada; 10 Department of Surgery, Western University, London, Ontario, Canada; Massachusetts Institute of Technology, UNITED STATES OF AMERICA

## Abstract

Surgical system decision-makers are confronted with the challenge of justifying investment in new surgical facilities against expanding the capacity of existing ones, and if worthwhile, where to place such new facilities. Geospatial analysis can provide findings to inform this decision. This scoping review examines how geospatial analysis has been used to identify geographically defined populations experiencing an inequitable access to surgical care to inform the care structures and processes. A comprehensive literature search was performed in November 2024 using the MEDLINE, EMBASE, Scopus, and PubMed databases for English-language peer-reviewed journal articles that carried out geospatial analysis of surgical care and identified geographic locations in need of resource allocations. Two reviewers independently screened studies for inclusion and extracted the data. Studies were summarized based on their findings and analytical methods. Of the nineteen studies included, three types of findings were presented: 1) two studies determined optimal locations for a new surgical facility; 2) six studies recommended strengthening the capacity of existing facilities after ruling out the need to build new ones, and 3) eleven studies identified locations that should be prioritized for resource allocation (e.g., more surgeons) without examining the need for new facilities or the possibility of strengthening existing ones. Gaps in the literature were identified to include a lack of explicit justification and identification of optimal sites for ambulatory surgical centers, use of dated registry data and potentially unreliable self-report data, and a lack of using the most appropriate geospatial techniques to answer surgical care planning questions according to the local context. Practical recommendations on geospatial analysis in surgical research are provided.

## Author summary

Geospatial analyses are performed to identify geographically defined populations with low access to health care. These results provide direct insights on how best to reallocate the scarce health resources or make new investments to mitigate the observed inequities in care access. Here we present a comprehensive review of English-language journal articles published between January 2010 and November 2024 to understand how geospatial analysis has been conducted to inform surgical care planning. Of the 19 studies we found, fourteen were conducted in a low- or middle-income country. When looking at the main conclusions, we found 2 studies determined specific optimal locations for a new surgical facility, 6 studies recommended strengthening the capacity of existing facilities after ruling out the need to build new ones, and 11 studies identified locations that should be prioritized for resource allocation (e.g., more surgeons) without examining the need for new facilities or the possibility of strengthening existing ones. These studies have used a wide range of geospatial techniques, which we summarized in this paper and discussed the strengths and pitfalls of each to provide guidance to researchers who may be interested in using these methods.

## Introduction

Disparities in surgical care is a global concern, costing an estimated 16.9 million lives annually [1], mostly in lower-middle income countries (LMICs) but also affecting high-income countries (HICs). For LMICs, accessing safe and affordable surgical and anesthesia care that are critical to population health remains a challenge according to the Lancet Commission on Global Surgery (LCoGS) [2]. In these resource-limited regions, surgical care planning prioritizes essential procedures such as the bellwether procedures (caesarean section, emergency laparotomy, and repair of open fractures) as hospitals that are equipped to perform these procedures are generally considered to have the minimum required infrastructure for other emergency surgery as well [3]. As such, the decision on where to direct resources to build bellwether-capable hospitals can have a profound impact on the local community. For HICs, the concern on surgical care accessibility is more on non-urgent or elective procedures, especially for marginalized or remote communities (such as rural residents) who live far away from the nearest provider and who may also have limited transportation options [4–6]. After witnessing hospitals being overburdened by unprecedented demand during the COVID-19 pandemic, some HICs (such as Canada [7], Australia [8] and the UK [9]) have started to reallocate low-complexity elective surgical cases away from the hospital to stand-alone ambulatory surgical centers. However, these decisions are met with criticism as investing in a new surgical center can be extremely costly and experts have expressed concerns on an accentuated socioeconomically based disparity in care access once these centers (especially privately owned ones) are in place [10]. Regardless of the type of jurisdictions, health care planners require information that can pinpoint where inequities in geographic accessibility of surgical care exist to guide investment and resources allocation decisions.

Geospatial analysis can provide insights to inform surgical care planning in both LMICs and HICs. Having emerged in the 1960s and initially focused on thematic mapping of populations and resources [11], geospatial analysis has evolved into powerful technologies like Geographic Information Systems (GIS), which create maps and analyze spatial relationships to inform city planning, environmental management, and disaster response [12]. In surgical care planning, geospatial analysis can be used to answer specific questions depending on the local context; for instance, decision-makers of LMICs may be interested in identifying which geographically defined populations have the poorest access to essential surgeries using 2-hour as a maximum travel time [2]; these insights can be used to guide surgical care capacity building (such as building new hospitals in underserved regions). For HICs, the interest may be to justify the need to open a new ambulatory surgical center based on the current geographic accessibility of a procedure (such as cataract surgery [10]) and precisely determine the optimal location to site this center. As such, choosing the most appropriate geospatial analytical techniques based on the local context and providing results that are relevant and actionable is crucial. Furthermore, as geospatial analysis often requires linkage of spatial and population data including many that are open-source, ensuring findability, accessibility, interoperability, and reusability (FAIR) [13] can increase the scientific rigor and policy relevance of this type of research.

In this scoping review, we assessed geospatial analyses published in last 15 years on surgery. As inequities in access to surgical care are known to be defined and measured differently in LMICs and HICs, our review aimed to capture studies in both settings. We sought to identify the type of research questions and conclusions examined by LMICs and HICs, summarized the techniques and data used, and provided a high-level synthesis on the strengths and limitations of different geospatial analysis methods. Results of this review have implications for a range of audience including researchers, health system planners, and decision-makers.

## Methods

We followed the Preferred Reporting Items for Systematic Reviews and Meta-analysis statement to conduct this scoping review (S1 Appendix) [14]. The review protocol was registered and publicly available on the Open Science Framework (https://osf.io/7xdkz/). We followed the iterative analytical frameworks outlined by Arksey and O’Malley [15] and further developed by Levac *et al*. [16]. The following questions were developed to guide this review study:

- What *research questions* have been addressed, and what *conclusions* did these studies reach according to the results of the geospatial analysis?
- What kind of *data* are required to conduct geospatial analyses focused on resource allocation?
- What were the *analytical techniques* used in these studies?

A literature search of English-language peer-reviewed journal articles on MEDLINE, EMBASE, Scopus, and PubMed was conducted on November 14, 2024 to find studies published from January 1, 2010 to the present. The search start date was picked to reflect a series of paradigm shifts in health geography research that occurred in the late 2000s to the early 2010s, including an increased ability to link diverse data to geospatial datasets, and the emergence of new techniques enabled by a dramatic improvement in computational power and the availability of open software [17,18]. One reviewer (RF), who is a health services researcher trained in surgical research, developed and executed a comprehensive search strategy for MEDLINE and adapted for the rest of the databases based on the search queries of published review studies [19,20] and in consultation with a medical geographer (PG) who has extensive experience examining the access to health care. Two concepts, including “spatial analysis” and “surgery”, were used by combining Medical Subject Headings (or Suggested Subject Terms) and free-text keywords (see search strategy in S2 Appendix). Manual checking of the reference list of each included study was performed by one reviewer (RF) in addition to a more thorough snowballing process [21] although no new studies were identified beyond the database searches.

We included articles that present an original investigation where geospatial analysis techniques were used to evaluate surgical care. All patient populations and surgical procedures were considered. We strictly required studies to produce actionable outputs by identifying a geographically defined population in need of more surgical care services or resources, such as a district of a city that would benefit from a new surgical facility. Studies were excluded for not using real-world data, unavailable in full text, only provided descriptive findings (such as maps), or did not pinpoint any geographically defined populations where reallocations of resources or establishment of new facilities may be needed based on study findings. Two reviewers (MC and RF) independently screened the titles, abstracts, and the full text on the Covidence platform. Disagreements were resolved through discussions.

The same reviewers developed and piloted tested two Excel forms to extract relevant data from the included studies. No significant changes were made to the data extraction tables upon pilot testing. The first form contained the following items: authors, publication years, country of data origin, study setting, type(s) of surgery and the facility (publicly funded, privately owned, or mixed), research objectives, main conclusions, and geographic locations identified for increased surgical resources. On the second form, we extracted the type of data collected, the analytical techniques used, and potential limitations or biases and any mitigation or justification by the authors. Studies were sorted by the country of data origin based on World Health Organization (WHO)’s six world regions [22]. We additionally classified countries into being high-, middle-, or low-income using the World Bank Atlas method based on gross national income per-capita in 2024 [23]. A thematic analysis was conducted to qualitatively summarize the included studies [24]. Microsoft Excel was used to organize the extracted data and calculate summary statistics.

## Results

### Study inclusion

**Fig 1:**
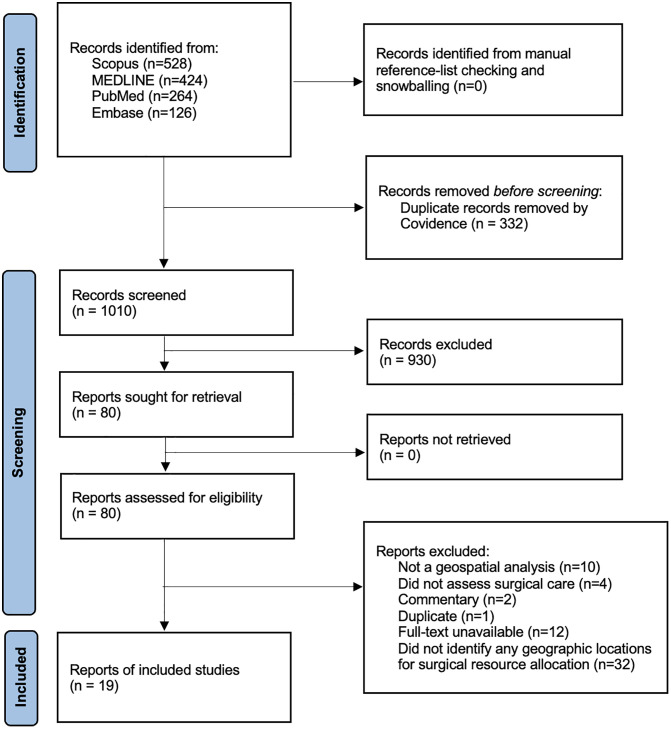
PRISMA diagram depicting study exclusion.

The initial database search yielded 1,342 citations, of which 1,010 were unique (Fig 1). No additional records were found through the manual reference-list checking and snowballing. Of these studies, 80 remained after the title/abstract screening. Upon full-text examination, we excluded 61 studies for the following reasons: not a geospatial analysis (n = 10), not assessing surgical care (n = 4), commentary (n = 2), duplicate (n = 1), full-text unavailable (n = 12) or did not identify a geographically based population for resource allocation (n = 32). As such, 19 studies were included in the review.

### Study characteristics

Of the 19 studies (Table 1), seven (37%) were based in the Africa Region (one each in the Democratic Republic of the Congo [25], Ethiopia [26], Ghana [27], Malawi [28], Nigeria [29], and two in Uganda [30,31]), another seven were based in the Region of the Americas (three in the USA [32–34], two in Brazil [35,36], one each in Guatemala [37] and Mexico [38]), two based in the Western Pacific Region (one each in New Zealand [39] and the Philippines [40]), and one each in South-East Asia (India [41]), Europe (the UK [42]), and Eastern Mediterranean (Somaliland [43]). Of these 15 countries, five were low-income (the Democratic Republic of the Congo, Ethiopia, Malawi, Uganda, Somaliland), four were lower-middle-income (Ghana, Nigeria, Philippines, India), three were upper-middle-income (Brazil, Guatemala, Mexico), and three were high-income countries (USA, New Zealand, the UK).

**Table 1:** Summary of basic study characteristics and main findings sorted by the WHO world region (n = 19).

Source	Country	Setting	Types of surgery and facility	Research objectives	Main conclusion on resource allocation	Where to allocate surgical resources?
**WHO Africa Region (n = 7)**						
Cairo 2020	Democratic Republic of the Congo (North Kivu)	40 facilities (32/33 health zones)	In-hospital pediatric (ped) surgery (Public/private)	To evaluate hospital capacity and variability in access to services	Limitations in access…are multifactorial with poor resources, few formally trained surgical providers, and near-absent access to pediatric anesthesiologists.	Enhancing the capacity for surgical care in and around the **health zone of Beni**
El Vilaly 2021	Nigeria	All qualifying health facilities (National)	In-hospital ped surgery (Public/private)	To identify the percentage of children who live within 2h of surgical care.	There is significant variation in surgical care access with a large majority of the population lacking access.	More ped surgeons and anesthetists are required in **rural areas outside the major urban population centres**
Endawkie 2024	Ethiopia	5527 reproductive-age mothers (National sample)	Caesarean section (Public/private)	To determine geospatial patterns and other factors of care delivery.	Caesarean section use remains below the WHO estimate with distinct geospatial variation.	Interventions are required to ensure fair access to caesaren section for **Tigray, Amhara, Afar, and Benishangul Gumze regions**
Farber 2016	Uganda	139 facilities (National)	In-hospital surgery (Public/private)	To determine the geographic distribution of unmet surgical disease burden.	There is a significant association between unmet surgical need and hub distance, area of coverage, and facility transport time.	Financial investment should be prioritized for the **Northern Sub-Region**
Smith 2017	Uganda	139 hospitals (National)	In-hospital ped surgery (Public/private)	To determine the distribution of surgical conditions among children.	Investing in surgery increases the rate of return for the entire health system.	Expansion of ped surgery should prioritize the **Northern region**
Stanton 2015	Malawi	476 hydrocelectomy cases (a single district hospital)	In-hospital hydrocelectomy (Public)	To assess the geographic distribution of hydrocelectomy and impact on quality of life.	There is an inverse association between distance to the hospital and the probability of having had a hydrocelectomy	**Rural areas** (such as **Ngabu**) may benefit from scaling-up of mobile mass surgery camps
Stewart 2016	Ghana	First- and second-level referral hospitals and all tertiary hospitals (National)	In-hospital bellwether procedures (Public/private)	To identify hospitals that would most improve access to essential surgery if strengthened.	There is a deficiency in essential surgical capabilities as well as spatial access gaps for a large percentage of the population.	Initiatives to strengthen surgical care should prioritize the **five ‘high-yield’ first-level referral hospitals (Comboni, Hawa Memorial Savior, Oda, Wiawso and Zebilla Hospitals)**
**WHO Region of the Americas (n = 7)**						
Buda 2022	Guatemala	37 surgery-performing and 9 non-surgical public hospitals (National)	In-hospital bellwether procedures (Public)	To evaluate access to surgery and identify hospitals where expansion of services would increase access.	Access to surgery is inadequate and varies regionally. Expansion of services in existing hospitals would minimally increase care access.	Adding facilities outside resource-concentrated area (such as **Baja Verapaz**)
Carrillo- Villasenor 2021	Mexico (Chiapas)	59 hospitals (statewide)	In-hospital bellwether procedures (Public/private)	To determine 2h access to hospitals capable of all bellwether procedures	Healthcare system fragmentation contributes to delays and unmet emergency surgical needs.	Enhance the bellwether capability of the **ten rural hospitals**
Han 2023	USA (California)	283 facilities (statewide)	In-hospital EGS (Public/private)	To determine the associations between EGS bypass behaviours and spatial access to EGS.	Resources were unequally distributed across regions, especially between rural and urban areas. Increased access to EGS could mitigate bypass behaviours.	Direct more and better quality EGS services to residents of **rural areas**.
Lee 2021	USA	All trauma centres and MTFs in continental US (National)	In-hospital trauma care (Public/private)	To determine which MTFs might best benefit populations by developing into trauma centres.	MTFs may benefit both military and civilian communities if developed into trauma centres.	**Seven MTFs** should be prioritized for developing level 1 or level 2 civilian-facing centres.
Nyaeme 2023	USA	941 counties in continental US (National)	Ambulatory otolaryngologic procedures (Public/private)	To assess clustering of utilization and the associated factors.	Ambulatory surgical centre use is the highest in cities which already have high levels of care access.	Government subsidies and incentives may target the **Midwest and upper Northeast**
Rocha 2020	Brazil	Total workforce (National)	In-hospital ped surgery (Public)	To illustrate ped surgical workforce in the public health system and identify associations with childhood mortality rates.	Investing in ped surgical care is required. Geospatial analysis can help define the workforce required to reach health goals.	Investment in surgical workforce should prioritize **rural regions.**
Vissoci 2019	Brazil	6498 district and referral level hospitals (National)	In-hospital ped surgery (Public)	To characterize surgical care delivery for children and examine the associated factors.	There are wide disparities in surgical care with respect to resources, infrastructure, and access to care.	Investment should target **hospitals in the North and Northeast** to meet the local demand for complex surgery
**WHO Western Pacific Region (n = 2)**						
Kruger 2012	New Zealand (Auckland)	442 medical and 256 dental practices (total in city)	Medical and dental practices (Public/private)	To determine if medical and dental practices are similarly distributed.	Inequalities exist with respect to geographic access and availability of primary dental care practices.	Efforts need to target the low density of primary adult dental practices for **outside the city centre (>2.5km)**
Lim 2023	Philippines	428 qualifying hospitals (National)	In-hospital bellwether procedures (Public)	To determine the proportion of population with 2h drive time to bellwether facility and identify future sites for a surgical facility.	Large disparities exist in timely access to facilities. Adding new hospitals in less accessible regions would improve access.	New bellwether-capable hospitals should be prioritized for **four regions (Caraga, Mimaropa, Calabarzon, and Zamboanga)**
**WHO South-East Asia Region (n = 1)**						
Dare 2015	India	565 district hospitals (National sample)	In-hospital diagnostics and treatment (Public)	To analyze spatial distribution of deaths from acute abdominal conditions and relate these to access to surgical facilities.	Focus on improving geographical access and existing hospital-level resourcing (human and physical resources).	Human and physical resources need to be strengthened at **existing government operated district hospitals**
**WHO European Region (n = 1)**						
Jo 2021	United Kingdom	4439 qualifying dental specialists (National)	Dental (Public/private)	To assess the geographic distribution of dental specialists in relation to population age and rurality.	Significant maldistribution of dental specialties exists. Expansion of the specialist workforce could alleviate the inequity in care access.	Planning and commissioning of the dental specialist workforce should prioritize **rural areas**
**WHO Eastern Mediterranean Region (n = 1)**						
Cotache-Condor 2021	Somaliland	1503 children and 15 hospitals (National sample)	In-hospital ped surgery (Public)	To analyze geographic distribution and access to ped surgical services.	Wide disparities in access should be addressed by investing in the expansion of surgical services and infrastructure to reduce geographical barriers.	Allocation of essential surgical care resources and interventions should target **Sahil and Sool**

Abbreviations: WHO, World Health Organization; ped, pediatric; min, minute; h, hour; EGS, emergency general surgery; MTF, military treatment facility.

Seven studies examined both adults and children [27,30,32,38,40–42], while six each focused on children [25,29,31,35,36,43] or adults [26,28,33,34,37,39]. Seven studies examined all surgery types [25,29–31,35,36,43], five focused on bellwether procedures [26,27,37,38,40], two examined dental procedures [39,42], and one each examined surgery for acute abdominal conditions [41], trauma surgery [32], emergency surgery [34], otolaryngologic procedures [33], and hydrocelectomy [28]. Twelve studies examined both publicly and privately owned surgical care facilities. The remaining seven studies focused on public hospitals, mainly government operated hospitals providing essential surgical procedures.

### Main conclusions on surgical care resource allocation

We summarized the research objective and conclusions of each study (Table 1). Three types of conclusions were identified, including 1) strengthening the resources of existing facilities after ruling out the need to build new facilities (n = 6); 2) building new facilities at optimal locations (n = 2); and 3) reallocating health resources to locations without assessing the need to enhance existing facilities or build new ones (n = 11). We summarized each type of conclusions below.

Six studies recommended strengthening the capacity of existing facilities instead of building new ones [25,27,32,36,38,41]. An Indian study [41] found by expanding human and physical resources at existing government operated district hospitals the disparity related to deaths from acute abdominal conditions due to poor access to surgery could be mitigated. A Ghanian study [27] identified five first-level referral hospitals to be prioritized for strengthening bellwether capacity. Similarly, a Mexican study [38] determined ten rural hospitals among the 59 hospitals in Chiapas where strengthening bellwether capacity would make the greatest impact on improving access to essential surgery. To embolden trauma care, a US study suggested prioritizing seven military treatment facilities for development into civilian-facing trauma centers [32]. A Congolese study [25] recommended enhancing the capacity of existing hospitals in the health zone of Beni to scale up pediatric surgical volume, and a Brazilian study [36] proposed prioritizing investment for existing hospitals in the North and Northeast to meet the demand for complex pediatric surgery.

Two studies identified optimal locations for a new hospital and both focused on bellwether procedures [37,40]. A Guatemalan study [37] found expansion of bellwether capability at existing public hospitals would provide minimal improvement of care access; as such, building new facilities in underserved regions (Baja Verapaz and El Progresso) may be warranted. The other study, conducted in the Philippines [40], determined four regions (Caraga, Mimaropa, Calabarzon, and Zamboanga City) where adding bellwether facilities would improve access to surgery.

Eleven studies identified geographic locations as priority targets for reallocating health resources without assessing the need to enhance existing facilities or build new ones [26,28–31,33–35,39,42,43]. Four studies [29,35,39,42] advocated for an increase in surgeons and anesthetists in rural areas. Five studies [30,31,33,34,43] suggested financial investments to target low-access regions; specifically, a US study [33] suggested that government subsidies and incentives provided to physicians and hospitals may reduce the low access to outpatient surgery observed in the Midwest and upper Northeast. A Malawian study [28] recommended scaling up mobile mass surgery camps for hydrocelectomy in rural areas. Another study [26] suggested interventions to be employed in four regions of Ethiopia to increase access to caesarean section without providing further details on such interventions.

### Data sources

The data sources were listed for each article (Table 2). Twelve articles obtained geospatial data from publicly accessible libraries, mostly commonly OpenStreetMap [25,27,29,37,38,43], Google Maps [27,32,39], and the WorldPop [25,27,28,37,40]. Sixteen articles [26–36,38–42] utilized administrative data to measure socioeconomic and health status, hospital locations, procedures, and costs; of them, thirteen studies [25,27–29,32,34–39,41,43] published between 2013 and 2022 used registry data collected before January 2020. Thirteen articles gathered data from surveys and censuses [25,26,28,30–34,37,39,41–43] to measure population sizes and other variables; three studies [28,30,31], all published before 2018, relied on self-reported travel times for the analysis. Six studies obtained data from other sources, including on-site data collection and personnel interviews [25,29,38,43], prior studies [41] and academic reports [39].

**Table 2:** Summary of analytical techniques and conclusions of the included studies by the WHO world region (n = 19).

Source	Data sources	Primary outcomes	Spatial analysis procedures (software used)
**WHO Africa Region (n = 7)**			
Cairo 2020	OpenStreetMap, WorldPop, the Humanitarian Data Exchange, Hospital audits and interviews	% population ≤ 2h travel to a facility with WHO safety score ≥ 8	• Random forests (AccessMod, SPSS) • Location-allocation analysis with 2h maximal travel time constraints
El Vilaly 2021	GRID3, ASTER Global DEM, OpenStreetMap, the Humanitarian Data Exchange, HydroSHEDS, Communication with anesthesia department heads and surgeons	% population ≤ 2h travel to facility with a pediatric surgical workforce	• Travel times calculated using Tobler’s Hiking Function • Accessibility analysis using a cost-weighted distance algorithm (ArcGIS)
Endawkie 2024	The DHS Program (containing data on population and regional distribution, socio-demographics, delivery characteristics, and maternal health service utilization)	Cesarean section use patterns	• Spatial distribution of care use • Spatial autocorrelation using Global Moran’s I • Hotspot analysis using Getis-Ord Gi to identify clustering • Regressions to assess factors associated with care use (QGIS, ArcGIS)
Farber 2016	SOSAS, Uganda’s Ministry of Health	Unmet surgical need patterns and association with geographic access	• 2SFCA • Spatial autocorrelation; Global Moran’s I; LISA to identify spatial clusters • SAR model used for spatial regression (ArcGIS, QGIS, GeoDa)
Smith 2017	(Same as Farber 2016, but analyzed USN in pediatric population)	(Same as Farber 2016, but analyzed USN in pediatric population)	(Same as Farber 2016, but analyzed USN in pediatric population)
Stanton 2015	Chikawa District Hospital operating theatre records, MASDAP, WorldPop	Association between surgical rates and distance to facility	Global Moran’s I and Anselin Local Moran’s I used to identify spatial clustering (ArcGIS, Microsoft Excel)
Stewart 2016	Ghana’s Ministry of Health, Direct contact with facilities & surgical logbook review, Google Earth, WorldPop, OpenStreetMap, CERSGIS	% population ≤ 2h travel to hospital with varied bellwether capabilities	Location-allocation identified facilities for improvement that would have the greatest effect on the population (ArcMap)
**WHO Region of the Americas (n = 7)**			
Buda 2021	AFAT, GPS, OpenStreetMap, NASA SRTM, Copernicus Global Land Service, WorldPop	% population ≤ 30 min, 1h, and 2h walking and driving times to facility	• Path Distance tool to estimate walk time; Tobler’s Hiking Function to account for vertical factor • Estimated increase in access once hospitals were equipped for essential surgical care (ArcGIS)
Carrillo-Villasenor 2021	DENUE, Cubos Dinamicos, SAT, OpenStreetMap, WorldPop, onsite interviews	% population ≤ 2h travel to a bellwether capable facility (WHO safety score ≥ 8)	Road network and road speeds used to calculate 2-hour access to facilities (Redivis)
Han 2023	AHA, California OSHPD PDD, ACS	Supply-to-demand ratio of EGS services for the local population	• Enhanced 2SFCA • Spatial autocorrelation using Global Moran’s I and BYM2 (R-INLA R package) • Gabriel Graph
Lee 2021	TCAA, ACS, MHS DMIS ID Tables, DHA, US Census Bureau-American Community Survey, Google Maps API	Estimated distance/time saved by converting some MTFs into civilian-facing trauma centres.	Harversine formula used to determine great-circle distances between points (Spyder)
Nyaeme 2023	CMS, US Census	Utilization of ambulatory surgical centre services by county	• Cluster analysis using Local Moran’s I (Python) • One-way ANOVA and t-test to identify factors associated with utilization
Rocha 2020	DATASUS, CNES, the World Bank, IBGE	• Associations between workforce and U5MR/POMR	• Choropleth maps used to display density of workforce and U5MR • Spatial autocorrelation within workforce density and U5MR depicted using Getis-Ord Gi • Regressions to identify associations between workforce and U5MR
Vissoci 2019	(Same as Rocha 2020)	(Same as Rocha 2020)	• Choropleth maps used to depict distribution of surgical delivery and U5MR • 2SFCA used to create an accessibility index (ArcMap) • Getis-Ord Gi used to evaluate spatial clustering of surgical delivery and mortality (ArcMap, GeoDa, QGIS) • Regressions to identify factors associated with care delivery and U5MR (ArcMap)
**WHO Western Pacific Region (n = 2)**			
Kruger 2012	Yellow Pages, Google Maps, New Zealand Census, Statistics New Zealand, Ministry of Health	% population ≤ 2.5-15 km or ≤ 2.5 km travel to a dental or medical practice	Analysis of geographic measures (ArcGIS)
Lim 2023	Department of Health (Philippines), GPS, WorldPop	% population ≤ 2h travel to a bellwether facility	• Random forests • Network analysis, via Dijkstra’s algorithm, to find the shortest path using 2h service area • Suitability modelling to determine the best location to site a facility (ArcGIS)
**WHO South-East Asia Region (n = 1)**			
Dare 2015	RGISRS, RHIME, MDS, DLHS-3, Global Rural-Urban Mapping Project, Commercial data vendor	Association between mortality clusters and proximity to well-resourced district hospitals	Spatial clustering analysis using the Getis-Ord Gi* method
**WHO European Region (n = 1)**			
Jo 2021	GDC specialist registrar, NHS Digital, HSC Business Services Organisation, and NHS National Services Scotland, English NHS CCGs, Scotland Health Boards, Wales Local Health Boards, and Northern Ireland Department of Health Trust, Office of National Statistics and UK Data Service Census	% population ≤ 2.5 km, 2.5-5 km, 5–10 km, & > 10 km travel to a dental specialist practice	• Geo-coded addresses and mapped over boundaries (QGIS, Microsoft Maps) • Analysis of population proximity to a specialist location; calculation of dental specialist ratio (Microsoft Excel)
**WHO Eastern Mediterranean Region (n = 1)**			
Cotache-Condor 2021	SOSAS, SAT & GAPS, GPS, OpenStreetMap	% child population ≤ 2h travel to a Bellwether facility	• Inverse-distance weighted interpolation to estimate condition burden • Hotspot analysis/Getis-Ord Gi*, Service area- Network Analyst, Voronoi diagrams (ArcMap)

Abbreviations: NASA, National Aeronautics and Space Administration; SRTM, Shuttle Radar Topography Mission; AFAT, Anesthesia Facility Assessment Tool; GPS, Global Positioning System; DENUE, Mexico’s Digital Statistical Directory of Economic Units; SAT, Surgical Assessment Tool; SOSAS, Surgeons Over Seas Assessment of Surgical Need; GAPS, Global Assessment in Pediatric Surgery; RHIME, Routine, Reliable, Representative, and Resampled Household Investigation of Mortality with Medical Evaluation; MDS, Million Deaths Study; DLHS-3, District Level Household and Facility Survey; GRID3, Geo-Referenced Infrastructure and Demographic Data for Development; ASTER, Advanced Spaceborne Thermal Emission and Reflection Radiometer; DEM, Digital Elevation Model; DHS, Demographic and Health Surveys; 2SFCA, two-step floating catchment area; ESDA, exploratory spatial data analysis; LISA, local indicators of spatial association; SAR, spatial autoregressive lag; AHA, American Hospital Association; OSHPD, Office of Statewide Health Planning and Development; PDD, Inpatient Discharge Data; AU, area unit; TCAA, The Trauma Center Association; ACS, The American College of Surgeons; MHS, Military Health System; DMIS, Defense Medical Information System; DHA, Defense Health Agency; MTF, Military Treatment Facility; CMS, Center for Medicare Services; DATASUS, Sistema Único de Saúde dataset; CNES, National registration of health establishments; IBGE, Brazilian Institute of Geography and Statistics; U5MR, under-5 mortality rate; POMR, perioperative mortality rate; MASDAP, Malawi Spatial Data Portal; CERSGIS, Centre for Remote Sensing and Geographic Information Services.

### Geospatial analysis techniques

The included studies applied a range of analytical methods (Table 2). When comparing studies conducted in LMICs to those in HICs, we did not identify any notable differences in the analytical methods used despite their largely different research objectives, population settings, and conclusions.

### Distance estimation

Two studies calculated the Enclidean distance between a population centre and its closest surgical centre as a measure of care access [30,31]. One study calculated the Euclidean distance between villages of patients and the facility where surgical care was actually delivered as a more realistic measure [28]. Another study applied incremental increases in Euclidean distance as a buffer around care providers [42]. One study used the Haversine formula to determine great-circle distances between points [32]. The Tobler’s Hiking function, which accounts for vertical factor, was used in two studies for walking times estimation [29,37]. One study used the Path Distance Tool to estimate walking time and the Network Analyst function to estimate driving time [37].

### Spatial accessibility analysis

Ten studies used some form of network analysis to measure access to care [25,27,30–32,34,36,38,40,43]. Four studies [30,31,34,36] employed the two-step floating catchment area method to create an accessibility index for each community based on time (or distance) required to travel to the nearest surgical care provider; areas with a low accessibility index were deemed to be candidates for increased care allocations. A more direct method to identify where best to allocate more resources was via a location-allocation analysis, as seen in two articles [25,27]. Other methods used to measure accessibility and identify low-access populations included suitability modeling [40] and service area analysis [25,43]. Visualization techniques were used to aid results presentation. Choropleth maps were used to show the distribution of population density [25,29,32,42], socioeconomic status [35,39], disease burden [30,35,41,43], travel times or distance [27,29–31,37,38,40,42], patterns of care utilization and available surgical care providers [26,28,30,31,33,35], and areas that should, in theory, reach a specific surgical care provider within a reasonable travel time [30,31,34,38,43]. Voronoi diagrams were used for assessing coverage of resources [27,34,43]. Two studies employed random forest-based dasymetric mapping to produce grid-level population density at fine-scale considering data such as land cover and use [25,40].

### Spatial association analysis

Ten studies conducted analyses to assess geospatial associations with care [26,28,30,31,33–36,41,43]. To assess patterns of clustering and dispersion, Global Moran’s I[26,28,30,31,34,36], Local Moran’s I[29–31,33,35], and Hot Spot Analysis using the Getis-Ord Gi* [26,35,36,41,43] were used. Interpolation was addressed by the Ordinary kriging technique [26] or an inverse distance weighted approach [43]. Regressions were conducted in four studies to identify associations between care accessiblity and population outcomes [30,31,35,36]. Other techniques used included an exploratory spatial data analysis (ESDA) and local indicators of spatial association [28,30,31,33]. ESDA was performed in two studies to evaluate whether spatial patterns of unmet surgical care needs were associated with the access to surgical centres [30,31].

### Software

ArcGIS was the most commonly used software [26,28–31,39,40], followed by the QGIS [26,30,31,36,42]. Other software used included the ArcMap [27,36,43], AccessMod [25], GeoDa [30,31,33,36], Google Maps API [32], Microsoft Excel [28,42], Microsoft Maps [42], Python [33], Redivis [38], RStudio [32], R-INLA [34], SaTScan [41], SPSS [25], and Spyder [32].

## Discussion

Geographic accessibility is a major barrier to timely surgical care according to the “three delays” framework (delay in seeking care, delay in reaching care, and delay in receiving care) [44]. This scoping review investigated the geospatial literature in the last 15 years whose analyses provided conclusions on where surgical care resources could be best allocated and how so. Of 19 included studies, two main findings emerged: 1) when it comes to providing findings to directly inform the establishment of new surgical care facilities or strengthening the capacity of existing ones, less than half of included studies (n = 8) did so. 2) Despite different research objectives and study settings, similar geospatial techniques were used; we highlighted the strengths and weaknesses of each technique to provide practical guidance for future research (Table 3).

**Table 3:** Evaluation of geospatial analysis techniques.

Purpose	Technique	Description	Advantages	Disadvantages
	Euclidean distance	Measures straight-line distance between two points	• Simple and easy to implement • Applicable across dimensions	• Locations may be inaccessible via straight-line travel (land obstacles or infrastructure)
**Distance estimation**	Haversine formula	Calculates great-circle distance between two points on a sphere given latitude and longitude	• Distance calculations are more accurate as the curvature of the Earth is considered • Suitable for use at the national level	• Assumes that two points lie on a sphere
	Tobler’s hiking function	Estimates cross-country walking speed over a sloped terrain	• Useful for populations where walking is the main mode of travel • Can link slope to travel time	• Assumes a walking speed of 6 km/h, thus the velocity gradient may vary over steeper slopes and longer distances
**Spatial accessibility analysis**	Two-step floating catchment area method	Measures physician to population ratio in two steps: Step 1) Using a prespecified catchment, calculate the service demand for each health facility. Step 2) Calculate an Accessibility Index for each population to reflect the amount of accessible services within its catchment	• Intuitive as it compares supply vs. demand • Suitable for identifying areas with low accessibility	• Assumes the population is evenly distributed and static over time • May omit poor access in impoverished inner-city communities
	Location-allocation analysis	Determines optimal sites for new facilities to maximize population coverage and/or minimize travel times	• Flexible to differing modes of travel • Can take into consideration the costs of travel • Directly identify optimal sites for new facilities	• The assumed travel scenarios may not be applicable to the whole population. • need to specify the number of new facilities before the analysis
	Service area analysis	Defines the area reachable within a given travel time or distance from a location	• Can help identify areas that lack access to essential services (both urban and regional) • Assists in optimizing emergency response based on real-world road networks • Able to account for different travel modes	• Neglects service demand and capacity • Assumes static network conditions (travel speeds and times) • Dependent on accurate and high-quality data • Computationally intensive for large networks
	Dijkstra’s algorithm	Calculates the shortest path from one point to every other point on a graph	• Simple and accessible for a wide range of applications • Guarantees optimal calculation of the shortest path	• Not applicable to graphs with negative weights • Performance can deteriorate with a high density of graph values
	Suitability modeling	Uses weighted criteria to rank the best locations for a particular use.	• Able to integrate multiple types of datasets • Flexible and customizable • Easy to interpret	• Weight assignment can be subjective, leading to bias in the map • Requires high-quality spatial data, leading to reduction in accuracy if there is any missing or outdated data • Potential for overfitting or misinterpretation if too many criteria are used
	Cost-weighted distance	• Calculates the least-cost path between points by considering the “costs” of travelling • Assigns a cost value to each type of terrain or obstacle • Other costs can include time, energy, and financial costs	• More realistic for practical applications • Adaptable to a wide range of scenarios • Provides more accurate data for planning and decision-making	• Can be computationally intensive and complex • Can be difficult to obtain the detailed data • Assigning cost values can be subjective • Cost surfaces require regular updates to reflect real-world changes
**Spatial association analysis**	Global Moran’s I	-Measures spatial autocorrelation based on both locations and values simultaneously • Evaluates whether a pattern is clustered, dispersed, or random	• Simple to interpret • Applicable to various types of spatial data and on a large scale	• Not as effective when the spatial pattern is not consistent across the study area • Difficulty distinguishing a random pattern from one with minimal deviation from the mean • Cannot discriminate between patterns of high and low values • May miss more local areas of dependence • Results sensitive to the defined spatial weights
	Getis-Ord Gi* (Hot Spot Analysis)	• Measures spatial clustering, via the G statistic, to assess association considering the concentration of weighted points • Evaluates the degree of clustering of high/low values	• Can discriminate between patterns of high and low values • Can identify local areas of dependence	• Not as effective when the spatial pattern is not consistent across the study area • Difficulty distinguishing a random pattern from one with minimal deviation from the mean • Results sensitive to the defined spatial weights • Values near the edges of the dataset could be inaccurate as there are fewer neighbors to influence the results
	Ordinary kriging	Spatial interpolation used to estimate unknown values at unmeasured locations based on spatial autocorrelation in a dataset.	• Accounts for spatial autocorrelation, allowing for more accurate predictions when data exhibit spatial dependence • Outputs variance maps, indicating confidence levels • Ensures unbiased predictions by assuming a constant mean	• Constant but unknown mean may not be applicable in cases where the trend varies • Time-consuming and computationally intensive, as it requires computing a semivariogram • Not ideal where abrupt changes in spatial data exist
	Spatial autoregressive lag model	Regression model with autoregressive errors and spatial lags of the dependent and independent variables	• Ideal in assessing high spatial autocorrelated data • Applicable where observations are influenced not only by their own characteristics but also the characteristics of neighboring observations	• Computationally intensive and complex, particularly for large datasets • Results are sensitive to the spatial weighting matrix • Assumes that the spatial dependence structure is homogenous across the entire study area
**Spatial association analysis (cont.)**	Geographically weighted regression	A local regression model for assessing spatially varying relationships using weights defined by spatial kernel function	• Considers spatial heterogeneity • Offers nuanced local insights of distinct variations and relationships • suitable for big dataset and large number of features	• Computationally intensive and complex • Interpretation more complex than global models
	Multi-level regression	Regression model used when data have a nested or hierarchical structure.	• Considers group-level dependencies • Handles both fixed and random effects, separating overall effects from group-specific variations (in-group vs. between-group variability)	• More computationally intensive than standard regression • Random effects can be difficult to interpret
	Exploratory Spatial Data Analysis	• A set of techniques used to visualize, describe, and summarize spatial data • Utilizes visualization techniques, descriptive statistics, spatial autocorrelation, hot spot and cluster analysis, and/or pattern point analysis	• Useful in the initial stages of data exploration and for hypothesis setting • Allows greater visual insight into complex spatial relationships and patterns • Provides more accurate data for planning and decision-making • Applicable at various scales	• Relies heavily on the quality of spatial data • Computationally intensive and requires technical expertise and knowledge of statistics, software, and/or programming • Results are sensitive to scale
**Spatial association analysis (cont.)**	Local Indicators of Spatial Association clustering	• Local variation of Moran’s I (also called Local Moran’s I) • Decomposes global measures of spatial association into the contributions of each observation, allowing for detection of local patterns (clusters, spatial autocorrelation, and spatial outliers)	• Allows for detailed insights into local spatial patterns • Detects outliers that may be masked by global measures	• Requires high-quality, fine-grained spatial data, which can be challenging and costly to obtain • Interpretation can be complex • Values near the edges of the dataset could be inaccurate as there are fewer neighbors to influence the results
	Inverse distance weighted interpolation	• Predicts values at unsampled locations based on values from nearby known locations • Assumes that points closer to each other are more alike than those further apart • Relies on distance decay, weight calculation, and an interpolation formula	• Simple to understand and implement • Allows local control over the interpolation by allowing adjustment of the power parameter	• Values near the edges of the dataset could be inaccurate as there are fewer neighbors available to influence the results • Assumes that spatial variation is the same in all directions • Accuracy highly dependent on the distance metric and power parameter

A persistent challenge faced by health care decision-makers is regarding if investing in new care facilities is worthwhile when compared to expanding the capacity of existing ones, and if so, where to place these new facilities. This decision is difficult in current times of budgetary constraints, as building a single-specialty ambulatory center with two surgical suites can cost between $2-$3 million US Dollars in HICs while a 350-bed district hospital in Africa with two operating theatres requires at least $4 million US Dollars annually to ensure basic operation [45]. In this review, we found 8 of the 19 included studies conducted their geospatial analysis to explicitly assess the need to build new facilities, and while six ruled out this option two determined optimal locations for new establishments. These practical findings and actionable data can directly inform investment in surgical infrastructure. We did not identify any HIC-based studies that identified optimal locations for new ambulatory surgical centers despite a growing appetite to invest in these facilities after the COVID-19 pandemic [8,9,46,47]; this gap needs to be addressed. Meanwhile, eleven of the 19 included studies did not explicitly examine the possibility of building new facilities or expanding the capacity of existing ones. Their conclusions were related to financial investments or other interventions for underserved regions. We suggest that when assessing how to mitigate inequity in geographic access of surgical care, researchers and health system planners should always conduct a geospatial analysis to first rule out the need for building new facilities before providing specific insights on how to strengthen existing facilities or deploy interventions for the entire region. Other limitations revealed by this review included use of a binary rural/urban variable to indicate the type of community [26,27,29–31,33,34,36–38,42,43]. To enable a more nuanced analysis that can demonstrate a socioeconomic gradient in surgical care access, use of multidimensional measures beyond simple indicators should be explored [48].

Three popular methods emerged from this review were spatial autocorrelation (Global Moran’s I), Hot Spot Analysis (Getis-Ord Gi*), and the Two-Step Floating Catchment Area (2SFCA) method (Table 3). The Global Moran’s I measures spatial autocorrelation and concludes if the patterns observed are clustered, dispersed or random [26,28,30,31,34,36]. The Getis-Ord Gi* statistic can more precisely identify statistically meaningful hot or cold spots[24,33,34,39,41. The 2SFCA method incorporates spatial data from the demand side (i.e., population sizes) and the supply side (e.g., number of physicians) to generate an accessibility index [30,31,34,36]. When used in conjunction with a location-allocation analysis [25,27], the 2SFCA can offer direct insights on where to place resources or new facilities. Since these geospatial techniques have distinct purposes of use, researchers need to avoid taking a ‘one size fits all’ approach as careful considerations on the local context and the strengths and pitfalls of each method need to be reflected in the study design, following the recommendation of the Lancet Commission on High-Quality Health Systems [49]. In this review, when comparing studies conducted in LMICs with those in HICs, the geospatial methods used were not notably different. Moving forward, we suggest researchers to identify locally meaningful research questions that align with the most urgent population health need, define the appropriate measure of equity and socioeconomic status, and incorporate characteristics of the population, cultural beliefs and other demographic and lifestyle factors to inform the selection of geospatial analytical method.

Our review results have important implications for both research and policy. The increased availability of open-source data (e.g., WorldPop) has enabled wide applications of geospatial analysis in resource-constrained settings including LMICs. These applications need to be supported by quality assurance efforts including ongoing data testing and use of a community-based approach to confirm these data align with the population [50]. The same requirements apply to self-reported data and facility registries as we found many studies in this review used dated or unreliable data, raising concerns on the reliability of their results. Next, novel data-driven methods are emerging, including machine learning-based travel time models using real-time data (such as mobile phone-based mobility data [51]). We did not identify any application of such methods in this review, which may imply an opportunity for future research. The validity of these methods needs to be established (such as by testing against patient logs) and potential concerns on data privacy ruled out before the application. Finally, based on our review, GIS-based approaches may enable the examination of optimizing the placement of new facilities or adjusting service levels at existing sites to match population demand. This information is invaluable for policymakers as they optimize the allocation of the scarce healthcare resources to improve the quality of care. A GIS that incorporates diagnostic and treatment data related to surgical conditions could enhance policy development, improve care quality and address unmet surgical needs. To achieve this, a standardized setup of GIS that includes concrete requirements on data layers, minimum resolution/acuity, and pathways of integrating the data insights into decision-making needs to be defined before the large-scale application [52].

Results of this scoping review need to be interpreted in the context of the study design. We restricted our search to be English-language studies which may have introduced language bias especially since most literature was based on non-English-speaking LMICs. Our focus on peer-reviewed journal articles may have also excluded considerations on grey literature including governmental reports. Furthermore, for the purposes of feasibility, we used a title-only search strategy in the Scopus and PubMed databases, which may have hindered a more comprehensive search and resulted in our relatively limited sample size (n = 19). Future review work should address these limitations by employing a more thorough search method; for instance, novel search tools enabled by artificial intelligence [53] may be leveraged while ensuring the highest rigor of research. Our scoping review design means that no formal appraisal on reporting quality of each included study was performed. Guidelines designed to assess the methodological quality of health geography research are emerging (such as the reporting checklist by the International Task Force for Spatial Lifecourse Epidemiology [54]) and may be used in the future.

## Conclusion

Geospatial analysis can provide actionable findings to directly inform the allocation of surgical care resources. This scoping review highlights 19 studies published in the last 15 years that have conducted a geospatial analysis to identify geographically defined populations reporting an inequitable access to surgery. We identified a paucity of studies that explicitly examined the possibility of building new surgical facilities and if worthwhile where to place such facilities. The included studies showcase the use of diverse analytical techniques and data sources although more careful consideration is warranted on identifying locally meaningful research questions and choosing the analytical method accordingly.

## Supporting information

S1 Appendix PRISMA checklist. (DOCX)

S2 Appendix Literature search and results. (DOCX)
