# Supplementary material for: Geospatial analysis in addressing surgical care inequities: A scoping review and methodological guide
Source: PLOS Digit Health. 2026 Jul 16;5(7):e0001510. doi: 10.1371/journal.pdig.0001510 (PMC13374894; doi:10.1371/journal.pdig.0001510)
Supplement: S2 Appendix — (DOCX) [file pdig.0001510.s002.docx]

**S2 Appendix Literature search and results**

Literature search was conducted on November 14, 2024

**Ovid MEDLINE: Epub Ahead of Print, In-Process & Other Non-Indexed Citations, Ovid MEDLINE® Daily and Ovid MEDLINE® <1946-Present>**

| **No.** | **Query** | **Hits** |
| --- | --- | --- |
| ***Concept #1 – Geospatial analysis*** | | |
| 1 | Geographic Information Systems/ or Geography/ | 53379 |
| 2 | Spatial Analysis/ or Spatial Regression/ | 6446 |
| 3 | ('geospatial' or (geospatial adj1 mapping) or (spatial adj1 distribution*)).ti,kf. | 14902 |
| ***Concept #2 – Surgery*** | | |
|  | General Surgery/ | 41167 |
|  | (surger* or surgical).ti,ab,kf. | 2374341 |
| ***Combined*** | | |
|  | 1 or 2 or 3 | 68828 |
|  | 4 or 5 | 2390032 |
|  | 6 and 7 | 598 |
|  | limit 8 to (English language and yr="2010 - Current") | 424 |

**EMBASE <1947 to current>**

| **No.** | **Query** | **Hits** |
| --- | --- | --- |
| ***Concept #1 – Geospatial analysis*** | | |
| 1 | *geographic mapping/ | 1060 |
| 2 | geographic distribution/ or geographic information system/ or geographic information/ | 200383 |
| 3 | *spatial analysis/ | 4002 |
| 4 | spatial autocorrelation analysis/ or spatial regression/ | 1772 |
| 5 | ('geospatial' or 'spatial' or 'map').mp. | 740842 |
| ***Concept #2 – Surgery*** | | |
| 6 | *surgical procedures, operative/ | 94478 |
| 7 | (surger* or surgical).ti,ab,kf. | 3174631 |
| ***Combined*** | | |
| 8 | ((1 or 2) and 3) or ((1 or 2) and (4 or 5)) | 17513 |
| 9 | 8 and (6 or 7) | 140 |
| 10 | limit 8 to (English language and yr="2010 - Current") | 126 |

**Scopus (Elsevier)**

| **No.** | **Query** | **Hits** |
| --- | --- | --- |
| ***Concept #1 – Geospatial analysis*** | | |
| 1 | TITLE (geospatial OR spatial) | 350638 |
| ***Concept #2 – Surgery*** | | |
| 2 | TITLE (surger* OR surgical) | 1425923 |
| ***Combined*** | | |
| 3 | AND [1-2] | 718 |
| 4 | Limit 3 to (published in 2010 or thereafter, English-language, and Articles) | 528 |

**PubMed**

| **No.** | **Query** | **Hits** |
| --- | --- | --- |
| ***Concept #1 – Geospatial analysis*** | | |
| 1 | geospatial [TITLE] OR spatial[TITLE] | 12107 |
| ***Concept #2 – Surgery*** | | |
| 2 | surger*[Title] OR surgical[Title] OR hospital*[Title] | 1157041 |
| ***Combined*** | | |
| 3 | 1 AND 2 | 461 |
| 4 | limit 4 to 2010 to present, English, and Humans | 264 |
